# Whole-genome sequence of *Salmonella enterica* subsp. *diarizonae* serovar 17: l,v:z: a new serovar isolated from a nose-horned viper in Republic of Serbia

**DOI:** 10.1128/mra.01174-24

**Published:** 2025-01-30

**Authors:** Kazimir Matović, Natasa Galić, Dejan Vidanović, Milanko Šekler, Marko Dmitrić, Nikola Vasković, Zoran Debeljak, Bojana Tešović, Aleksandar Simović, Ivan Bošnjak, Dejan Krnjaić, Jörg Linde, Ulrich Methner

**Affiliations:** 1 Veterinary Specialized Institute Kraljevo, Kraljevo, Serbia; 2 Institute of Public Health of Serbia Dr Milan Jovanovic Batut, Belgrade, Serbia; 3 Serbian Herpetological Society Milutin Radovanović, Belgrade, Serbia; 4 Academy of Applied Preschool Teaching and Health Studies Kruševac—Ćuprija Department, Ćuprija, Serbia; 5 Faculty of Veterinary Medicine, University of Belgrade, Belgrade, Serbia; 6 Institute of Bacterial Infections and Zoonoses, Friedrich-Loeffler-Institute, Jena, Germany; Montana State University, Bozeman, Montana, USA

**Keywords:** *Salmonella*, new serovar, antimicrobial resistance, snake, nose-horned viper, whole-genome sequence, *Salmonella enterica* subsp. *diarizonae*

## Abstract

We report the draft genome sequence of *Salmonella* enterica subsp. *diarizonae* serovar 17: l,v:z isolated from a nose-horned viper (*Vipera ammodytes*) in Republic of Serbia which might be considered a new serovar of *Salmonella*.

## ANNOUNCEMENT

Salmonellosis is an infectious disease caused by bacteria of the genus *Salmonella*. Infections or diseases by *Salmonella* organisms occur in both animals and humans and represent one of the keymost important zoonoses. The two species in the genus *Salmonella* (*S*.) are *S. enterica* and *S. bongori*.

Here, we report the whole-genome sequence of *Salmonella* enterica subsp. *diarizonae* serovar 17: l,v:z isolated from a nose-horned viper (*Vipera ammodytes*) in the Republic of Serbia which might be considered a new serovar of *Salmonella*. The strain was sampled on 27 July 2012 in a study aimed to examine the occurrence of *Salmonella* organisms in snakes/poisonous snakes in the place of Milosev Do (geolocation 43.31867024, 19.783844), Prijepolje municipality, Republic of Serbia. Primary laboratory processing of samples (isolation) and molecular confirmation of Salmonellae (Real-Time PCR) was performed in the laboratory of the Veterinary Specialized Institute Kraljevo, in Kraljevo (1, 2). The *Salmonella* isolate was referred to the Institute of Public Health of Serbia Dr Milan Jovanovic Batut, Belgrade, Republic of Serbia for biotyping and serotyping. The results showed the presence of new serotype, that is not listed in the White-Kauffmann-Le Minor scheme (3), with the antigenic formula 17: l,v:z.

The isolate was also sent to “Institut Pasteur” (International Center for Reference on *Salmonella*), a letter by the Head of the Enteric Bacterial Pathogens Unit (Franois-Xavier Weill) confirmed that the *Salmonella* strain 17: l,v:z might be considered a new serovar (Test report dated 7 July 2017).

Isolation of *Salmonella* was carried out as a procedure for the analysis of fecal samples according to ISO 6579-1 (4). Serotyping was performed according to the White-Kauffmann-Le Minor scheme (3).

Antimicrobial susceptibility of the strain was assessed by determining the minimum inhibitory concentration (MIC) using the broth microdilution method with Sensititre TM EUVSEC plates (Trek Diagnostic Systems Ltd., East Grinstead, United Kingdom). Epidemiological cut-off values were used according to the European Committee on Antimicrobial Susceptibility Testing (EUCAST) (5). The MIC values (µg/mL) of the *Salmonella* strain with the antigenic formula 17: l,v:z for each antimicrobial were as follows: 128 (sulfamethoxazole), <0.25 (trimethoprim), 0.03 (ciprofloxacin), 4 (tetracycline), 0.03 (meropenem), 16 (azithromycin), 8 (nalidixic acid), <0.25 (cefotaxime), <8 (chloramphenicol), 1 (tigecycline), 0.05 (ceftazidime), <1 (colistin), <1 (ampicillin), and 1 (gentamicin). Therefore, the strain does not reveal any antimicrobial resistances.

Short-read sequencing, genome assembly, and genome analysis were performed as previously described (6). In short, the QIAGEN Genomic-tip 20/G kit (QIAGEN, Germany) was used to prepare DNA. The Nextera XT DNA Library Preparation Kit (Illumina Inc., USA) was used to create libraries which were paired-end sequenced (2*300 bp) on an Illumina MiSeq device (Illumina Inc., USA). After quality control with FastQC (7), raw reads were assembled using Shovill v. 1.0.4 (8), with SPAdes as assembler (9) and Pilon v.1.24 (10) for error polishing. QUAST v. 5.0.2 (11) was used to assess the quality of assembled genomes and Kraken v2.1.1 (12) to screen for contaminations. The software mlst v. 2.16.1 (13) detected Multilocus-Sequence-Types. AMRFinderPlus (v. 3.6.10) was used (14) to scan for genetic factors for antimicrobial resistance and the Virulence Factor Database (15) for genes coding for virulence factors. 84% of the 1.430.614 raw reads reached Q30, while 82% belonged to the genus *Salmonella*. The reads were assembled into a 5.8 MB genome (N50 137.840 bp, GC 51,22%) containing a plasmid with a ColRNAI_1 replicon. The predicted serovar of SeqSero2 (16) was IIIb 17: l,v:z while SISTR (17) predicted IIIb 17(J):l,v:z including a warning (“only matched 169 cgMLST330 loci”). Multilocus-Sequence-Typing using the scheme “senterica_achtman_2” detected Sequence Type 1156. While 122 genes coding for potential virulence factors were detected, the genome contained no genetic factors for antimicrobial resistance.

## Data Availability

The raw sequencing data of *Salmonella enterica* subsp. *diarizonae* serovar 17: l,v:z strain 23PM0060 were submitted to the European Nucleotide Archive (ENA) and is available under the accession number PRJEB81563.
